# Optogenetic auditory fMRI reveals the effects of visual cortical inputs on auditory midbrain response

**DOI:** 10.1038/s41598-018-26568-1

**Published:** 2018-06-07

**Authors:** Alex T. L. Leong, Celia M. Dong, Patrick P. Gao, Russell W. Chan, Anthea To, Dan H. Sanes, Ed X. Wu

**Affiliations:** 1 Laboratory of Biomedical Imaging and Signal Processing, The University of Hong Kong, Pokfulam, Hong Kong SAR, China; 2 Department of Electrical and Electronic Engineering, The University of Hong Kong, Pokfulam, Hong Kong SAR, China; 3 Center for Neural Science, New York University, New York, NY 10003 United States; 4 School of Biomedical Sciences, Li Ka Shing Faculty of Medicine, The University of Hong Kong, Pokfulam, Hong Kong SAR, China; 5 Department of Medicine, Li Ka Shing Faculty of Medicine, The University of Hong Kong, Pokfulam, Hong Kong SAR, China

## Abstract

Sensory cortices contain extensive descending (corticofugal) pathways, yet their impact on brainstem processing – particularly across sensory systems – remains poorly understood. In the auditory system, the inferior colliculus (IC) in the midbrain receives cross-modal inputs from the visual cortex (VC). However, the influences from VC on auditory midbrain processing are unclear. To investigate whether and how visual cortical inputs affect IC auditory responses, the present study combines auditory blood-oxygenation-level-dependent (BOLD) functional MRI (fMRI) with cell-type specific optogenetic manipulation of visual cortex. The results show that predominant optogenetic excitation of the excitatory pyramidal neurons in the infragranular layers of the primary VC enhances the noise-evoked BOLD fMRI responses within the IC. This finding reveals that inputs from VC influence and facilitate basic sound processing in the auditory midbrain. Such combined optogenetic and auditory fMRI approach can shed light on the large-scale modulatory effects of corticofugal pathways and guide detailed electrophysiological studies in the future.

## Introduction

Sensory cortices contain extensive descending (corticofugal) projections to subcortical nuclei^1^ and widespread connectivity among different sensory systems^2–4^. While the impacts of the corticofugal input on brainstem information processing remain poorly understood, it is plausible that they have cross-modal influences, to enhance detection of and responses to salient external stimulation^5,6^. In the auditory system, a major target of the corticofugal projections is the inferior colliculus (IC) in the midbrain, which integrates ascending information from multiple brainstem nuclei^7^. The IC receives dense innervation from the auditory cortex (AC)^8–11^ and cross-modal inputs from other sensory cortices, such as the visual cortex (VC)^12–16^. While both the auditory and visual cortices can impact auditory midbrain processing immensely, existing studies have predominantly investigated AC influences^17,18^. Despite the known cortical interactions between these two sensory systems^3–5,19–21^, cross-modal influences from VC on auditory midbrain processing remain unclear.

Functional magnetic resonance imaging (fMRI) provides the most versatile imaging platform for mapping the brain activities *in vivo*^22–24^. Basic and clinical researchers utilize fMRI to map local brain functions via measuring large-scale neural activations throughout the brain in response to sensory stimulation or cognitive tasks in health and disease states. By analyzing the blood-oxygenation-level-dependent (BOLD) signals in response to the task, large-scale activations at different local brain regions can be robustly and reliably detected in animals and humans. Our initial attempt to reveal these cross-modal influences from VC employed BOLD fMRI^23^ technique to examine the auditory responses in rat IC after bilateral ablation of either the visual or auditory cortex^25^. The results indicated that VC ablation decreases IC responses to noise stimulation significantly, an effect opposite to that of AC ablation, suggesting that VC facilitates IC responsivity. Although our results provided evidence for the large-scale cross-modal influences from VC on auditory midbrain processing, further dissection of such cross-modal influences is not feasible with the gross cortical ablation manipulation.

Existing methodologies to study corticofugal functions, including our previous study, have limitations. Specifically, most studies have employed non-specific cortical modulation, such as electrical stimulation^26–29^ or chemical/cryogenic deactivation^30–32^. These cortical manipulations affect all cortical neuron types in the stimulation region, even though descending projections arise primarily from pyramidal neurons^17,18^. In addition, it is difficult to extrapolate the coordinated activity across all IC subnuclei using single neuron recordings^8–11^. Moreover, although it has been reported that visual stimulation can modulate auditory responses in IC neurons^33,34^, and that a subset of IC neurons can respond to visual stimulation alone^35–37^, these studies were unable to examine the extent of large-scale modulatory effects of visual inputs on IC. Specifically, they were unable to determine the effects of inputs from VC because visual stimulation was unspecific and activated all visual regions. These drawbacks preclude a comprehensive and specific investigation of corticofugal functions at large-scale^38,39^.

Optogenetics is an emerging technique that provides cell-type specific, millisecond-scale and reversible neuromodulation by expressing light-sensitive microbial opsin proteins in genetically targeted neurons with minimal invasiveness^40–45^. Recently, it has been combined with fMRI to measure the causal generation of BOLD signals following activation of specific excitatory neurons^46–48^ or probe the functional dynamics in large-scale brain networks^49–53^. Here, by further integrating direct sensory stimulation such as an auditory stimulus, we propose to deploy optogenetic fMRI to interrogate the role of cortical descending inputs in sensory processing.

In this study, we examined the effects of optogenetic stimulation of VC on the baseline and sound-evoked BOLD fMRI signals in the auditory midbrain. Specifically, the optogenetic stimulation targeted the excitatory pyramidal neurons in the infragranular layers (i.e. layers V & VI) of the primary VC, which constitute a major source of corticofugal projections^54,55^. We show that optogenetic activation of the VC enhances the sound-evoked BOLD responses in the IC. This indicates that inputs from the VC exert facilitatory influence on the IC for sound processing, and such effect is likely driven by the excitatory neurons in the infragranular layers. Our results demonstrate the feasibility and promises of this optogenetic auditory fMRI approach for investigating the large-scale cortical descending influences on auditory midbrain processing.

## Methods

### Animal Subjects

All animal experiments were approved by the Committee on the Use of Live Animal in Teaching and Research of the University of Hong Kong. Twenty adult Sprague-Dawley male rats were used as subjects. The sample sizes of all animal experiments are outlined in Table 1. They were housed under a 12-hour dark-light cycle with access to food and water *ad libitum*. Note that each animal underwent both VC optogenetic/blue light stimulation only and combined auditory and VC optogenetic/blue light stimulation.

**Table 1:** Sample size for each experiment group (optogenetic and naïve animals).

Experiment Group	10 Hz VC Optogenetic Stimulation	1 Hz VC Optogenetic Stimulation	10 Hz VC Blue Light Stimulation	1 Hz VC Blue Light Stimulation
Sample Size	9	2	5	4

Note that each animal underwent both VC optogenetic/blue light stimulation only and combined auditory and VC optogenetic/blue light stimulation.

### Optogenetic Stimulation Setup

#### Virus Packaging

Recombinant adeno-associated virus expressing a Channelrhodopsin2-mCherry fusion protein under control of the Ca^2+^/calmodulin-dependent protein kinase IIα (CaMKIIα) promoter was used. The AAV5-CaMKIIα-ChR2(H134R)-mCherry plasmid (map available online from www.stanford.edu/group/dlab/optogenetics) was packaged by the viral vector core of the University of North Carolina at Chapel Hill, Chapel Hill, NC (titre of 4 × 10^12^ particles/mL).

#### Viral Injection

**Figure 1:**
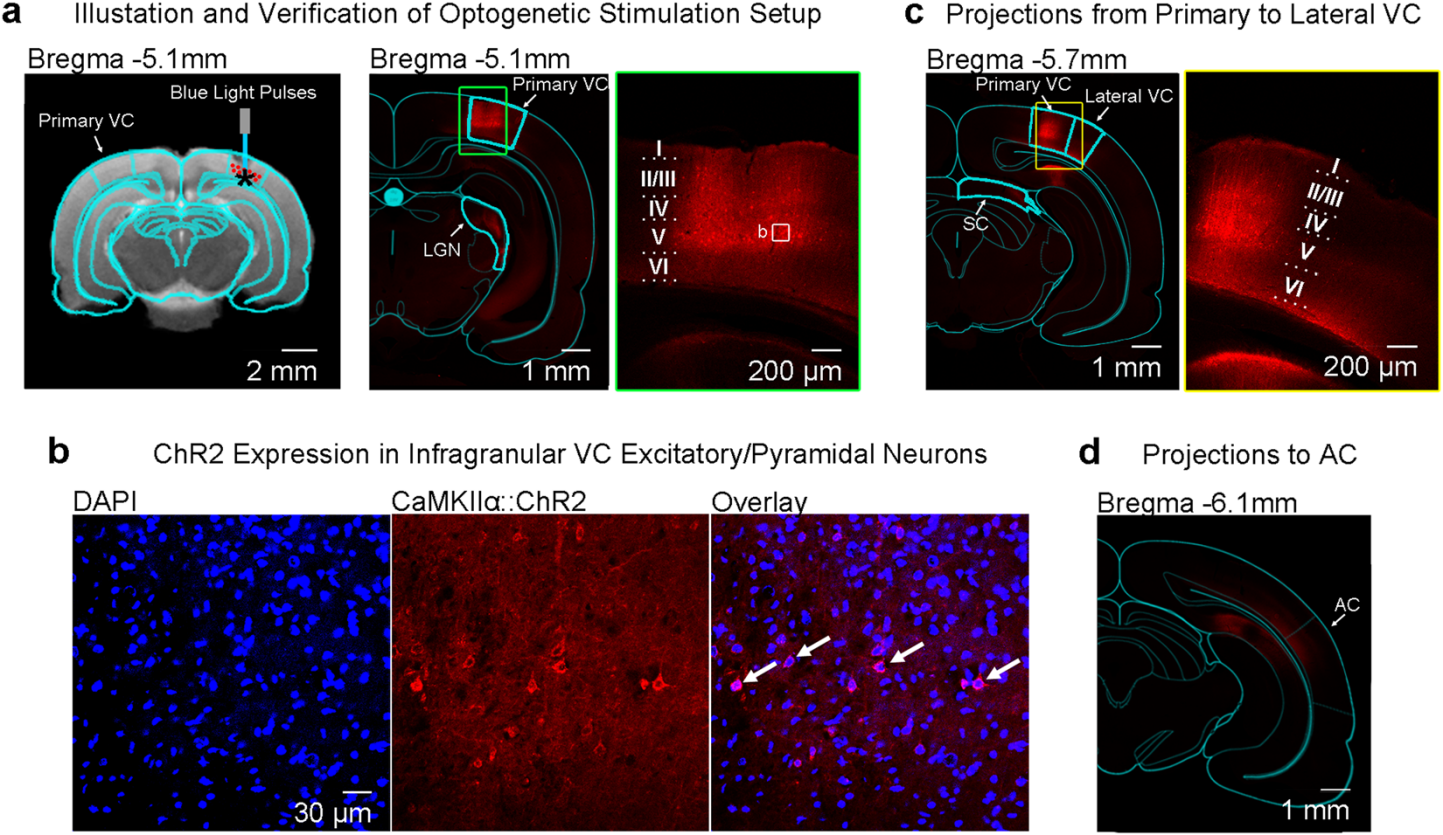
Optogenetic stimulation setup and histological characterization of ChR2::mCherry expression in the excitatory pyramidal neurons of primary visual cortex (VC). (**a**) Left: illustration of AAV (indicated by red dots) injection and fiber (indicated by blue line) implantation site in the right primary VC; Middle: Low-magnification confocal image showing ChR2 expression in primary VC and the lateral geniculate nucleus (LGN); Right: High-magnification confocal images showing ChR2-mCherry expression concentrated in the infragranular layers of primary VC, particularly layer V. The box indicates the area magnified in (**b**). (**b**) Overlay of images co-stained for the nuclear maker DAPI (blue) and mCherry (red) revealing co-localization of mCherry, in the cell bodies of infragranular pyramidal excitatory neurons (indicated by arrows). Note the characteristics of infragranular excitatory neurons, which are large and pyramid in shape. (**c**) Left: Low-magnification confocal image showing the ChR2 expression in primary VC to lateral VC projections and the corticofugal projections to SC. Right: High-magnification confocal image showing the ChR2 expression in primary VC to infragranular lateral VC projections. (**d**) Low-magnification confocal image showing the minute ChR2 expression in primary VC to infragranular AC projections. Note that the confocal image was brightened 1.5x to better visualize ChR2 expression patterns in the projections to AC.

Stereotactic surgery was performed when rats were 6–7 weeks old (~250 g). Rats were anesthetized with an intraperitoneal bolus injection of ketamine (90 mg/kg) and xylazine (40 mg/kg) mixture. The scalp was shaved, and the rats were secured in a stereotactic frame. Buprenorphine (0.05 mg/kg) was administered subcutaneously to minimize pain and heating pads were used to prevent hypothermia. Following a midline incision, a craniotomy was made on the right hemisphere in the primary VC and injection was performed at two depths (−5.5 mm posterior to Bregma, +3.8 mm ML, −1.0 and −1.5 mm from brain surface, Fig. 1). For optogenetic animals, 1.5 μL of viral constructs were delivered through a 5 μL syringe and 33-gauge bevelled needle injected at 150 nL/min at each depth. For naïve animals, 1.5 μL of saline was injected instead of the viral constructs at each depth. Following injection, the needle was held in place for 10 minutes before slow retraction. Then, the scalp incision was sutured. After the surgery, buprenorphine (0.05 mg/kg) was administered subcutaneously twice daily for 72 hours to minimize discomfort. Enrofloxacin was administered orally for 72 hours to minimize post-surgery infection and inflammation. Animals rested for 4 weeks before fMRI experiments were performed.

#### Fibre Implantation

Stereotactic surgery was performed to implant a custom-made plastic optical fibre cannula (POF, core diameter 450 μm; Mitsubishi Super ESKA^TM^ CK-20) at the injection site 1–2 hours before fMRI experiments. Rats were anesthetized with isoflurane (induction 3% and maintenance 2%) and secured on a stereotactic frame. Following a midline incision, a craniotomy was made at the same coordinates as the injection site. Before implantation, the fibre tip was bevelled to facilitate insertion and minimize injury to brain tissue. Then, it was inserted with the fibre tip at depth of 1.5 mm, which approximately corresponds to layer VI of primary VC (verified for each animal by anatomical MRI before fMRI sessions). Dental cement fixed it on the skull, and scalp incision was sutured. The fibre outside brain was made opaque using heat shrinkable sleeves to avoid undesired visual stimulation. After the surgery, buprenorphine (0.05 mg/kg) was administered subcutaneously to minimize discomfort.

#### Light Stimulation

The light stimulation was controlled by a computer, produced by a DPSS laser (blue light with 473 nm wavelength) and delivered into the bore of the MRI magnet using an optical patch cable (5–10 m) connected to the POF.

### Auditory Stimulation Setup

Auditory stimulation was controlled by a computer and produced by a high frequency magnetic speaker (MF1, TDT) driven by an amplifier (SA1, TDT). Monaural stimulation was delivered through a custom-made 165 cm long rigid tube and a 6.5 cm soft tube into the animals’ left ear. The right ear was occluded with cotton and Vaseline, to reduce the acoustic noise of scanner reaching the ears. The occlusion attenuated sound by approximately 40 dB. This setup has been used in our previous studies^25,56–59^.

### Animal Setup for fMRI

For fMRI experiments, animals were mechanically ventilated via oral intubation. Then they were placed on a stereotactic holder in the prone position with a tooth bar to restrict head motion. Throughout the course of MR scanning, anaesthesia was maintained with 1.0% isoflurane, and warm water was circulated. Animal heart rate, respiration rate, oxygen saturation and rectal temperature were continuously monitored by sensors (SA instruments) and kept in normal ranges (heart rate: 380–420; respiration rate: 56–60; oxygen saturation: >95%; rectal temperature: 36.5–37.5 °C). The preparation here was similar to our previous studies^25,56–67^.

### fMRI Data Acquisition

**Figure 2:**
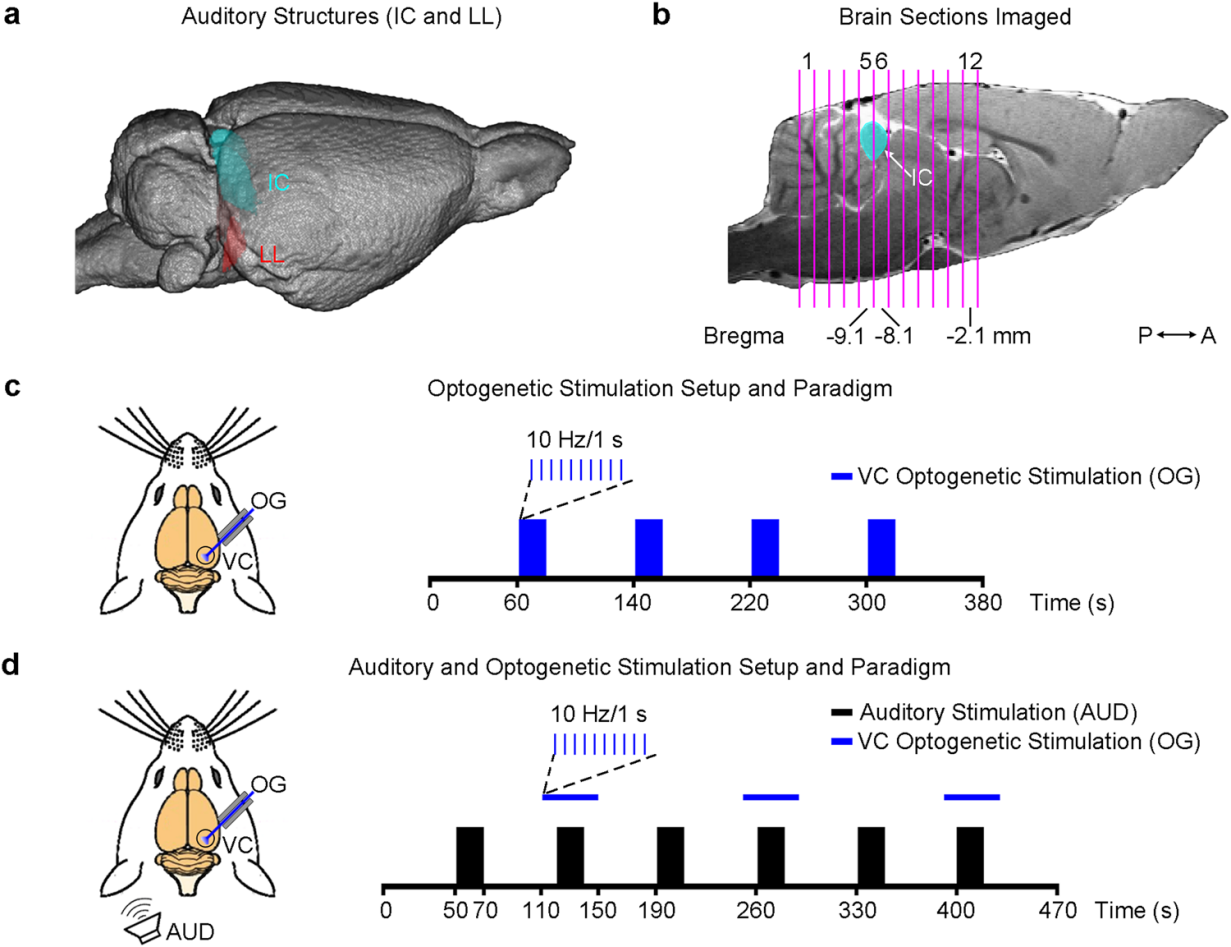
fMRI data acquisition and optogenetic/auditory stimulation paradigms. (**a**) Illustration of the inferior colliculus (IC) on a 3D MRI rendered brain. The lateral lemniscus (LL), a subcollicular auditory nucleus^7^, is also shown. (**b**) The location of 12 coronal brain sections (the 5^th^ and 6^th^ covering the center of the IC) imaged in this study. (**c**) Optogenetic stimulation (OG): illustration of the setup (left) and the block-design paradigm used to present the stimulation (right). Blue light pulses (light wavelength: 473 nm, pulse rate: 10 Hz, pulse duty cycle: 10%, light intensity: 40 mW/mm^2^) were presented to the right visual cortex (VC) in blocks of 20 s light-on and 60 s light-off. The paradigm was repeated twice in each animal. (**d**) Combined auditory (AUD) and optogenetic (OG) stimulation: illustration of the setup (left) and the block-design paradigm used to present the bi-modal stimulation (right). A broadband noise (bandwidth: 1–40 kHz; sound pressure level: 90 dB) was presented to the left ear of animals in blocks of 20 s sound-on and 50 s sound-off, while blue light pulses were presented to the right VC from 10 s before to 10 s after, during every even sound-on period. This paradigm was repeated 4 times in each animal.

All MRI experiments were performed on a 7 T MRI scanner (PharmaScan 70/16, Bruker Biospin GmbH) using a transmit-only birdcage coil in combination with an actively decoupled receive-only surface coil. Scout images were first acquired to determine the coronal and sagittal planes of the brain. 12 coronal slices with 1.0/0.0 mm thickness/gap were positioned to cover the auditory pathway with the 5^th^ and 6^th^ on the IC (at Bregma −9.1 mm and −8.1 mm, Fig. 2a,b). T2 weighted images were acquired as anatomical reference using a Rapid Acquisition with Refocused Echoes (RARE) sequence (FOV = 32 × 32 mm^2^, data matrix = 256 × 256, RARE factor = 8, TE/TR = 36/4200 ms). Then fMRI measurements were obtained using a multi-slice single-shot Gradient-Echo Echo-Planar-Imaging (GE-EPI) sequence (FOV = 32 × 32 mm^2^, data matrix = 64 × 64, flip angle = 56°, TE/TR = 20/1000 ms, temporal resolution = 1000 ms).

### Optogenetic and Auditory Stimulation Paradigms

The effects of optogenetic stimulation in the VC on brain baseline BOLD signals were examined by presenting blue light pulses (light wavelength: 473 nm, intensity: 40 mW/mm^2^, pulse rate: 10 Hz and 1 Hz, duty cycle: 10%) in a block-design paradigm (60 s light-off followed by 4 blocks of 20 s light-on and 60 s light-off, fMRI no. of time points = 380), without presenting sound stimulation (Fig. 2c). This paradigm was repeated twice in each animal. Subsequently, the effects of optogenetic stimulation on auditory midbrain processing of sound stimulation were investigated. A broadband noise (bandwidth: 1–40 kHz; sound pressure level or SPL: 90 dB) was presented to the left ear of animals in a block-design paradigm (50 s sound-off followed by 6 blocks of 20 s sound-on and 50 s sound-off, fMRI no. of time points = 470). The optogenetic stimulation (light wavelength: 473 nm, intensity: 40 mW/mm^2^, pulse rate: 10 Hz and 1 Hz, duty cycle: 10%) was presented to the right VC from 10 s before to 10 s after every even sound-on period (Fig. 2d). The off period was reduced to 50 s to shorten the total scanning time and this paradigm was repeated 4 times in each animal. Other pulse rates were also examined but not presented here, as they did not significantly modulate the auditory midbrain responses examined by the present study. Before each experiment, both the optogenetic and auditory stimulation were calibrated outside the magnet room. The light power at the fibre tip was measured using a power meter (PM100D, Thorlabs, USA). The sound waveform was measured by a recorder (FR2, Fostex, Japan) placed at ~2 mm from the tip of the flexible tube. The variance of the light power was maintained less than 2.5 mW/mm^2^ and the noise SPL less than 2 dB^25,56,57^.

### fMRI Data Analysis

In each experiment, the fMRI images from each animal were realigned to the mean image of the first fMRI session (SPM8, Wellcome Department of Imaging Neuroscience, University College London, UK). Images from different animals were co-registered to a custom-made brain template using affine transformation and Gaussian smoothing, with the criteria of maximizing normalized mutual information (SPM8). Linear detrending was then performed voxel-wisely. Data from repeated sessions were averaged, in-plane smoothed (FWHM = one pixel) and high-pass filtered (128 s), and then standard general linear model (GLM) was applied^25,56–59^ to calculate the BOLD response coefficient (β) maps for each stimulus (SPM8). Typically, in each animal, two fMRI sessions were averaged for VC optogenetic/blue light stimulation only whereas four sessions were averaged for combined auditory and VC optogenetic/blue light stimulation. Finally, activated voxels were identified with following Student’s t test on the β values (p < 0.05, corrected for FWE).

Three regions-of-interest (ROIs) covering different IC subdivisions were defined using the Paxinos & Watson rat brain atlas^25^. The ROI that covered VC, RS or SC was defined by identifying clusters of activated voxels (p < 0.05, corrected for FWE) that were restricted within the anatomical location of each region. Anatomical locations of VC, RS and SC were determined using the atlas. In individual animals, the BOLD signal profiles for each ROI were first extracted and averaged across voxels, before they were separated into six blocks (each covering a period from 10 s before to 30 s after a sound-on period) and four blocks (each covering a period from 10 s before to 50 s after a optogenetic-on period), respectively. They were then averaged again, and normalized by the mean signal intensity of the first 10 s to calculate the percentage of BOLD signal change. Final averaging was then performed across animals to generate BOLD signal profiles.

Additionally, in individual animals, β values were also extracted from each ROI and averaged across voxels. The final β value used for comparisons between the IC BOLD responses to the broadband noise stimulation with and without optogenetic stimulation of the VC was computed by averaging. Note that the size of ROIs for each IC subdivision was different and this could influence the absolute SNR of the averaged BOLD responses.

### Histology, Immunohistochemistry and Confocal Imaging

Upon completion of the fMRI experiments, animals were deeply anesthetized with pentobarbitol and transcardially perfused with ice-cold 4% paraformaldehyde (PFA) in PBS. The brains were equilibrated in 20% sucrose in PBS at 4 °C overnight. Axial brain sections (40 μm) were prepared on a freezing microtome (model #860, AO scientific instruments). Consecutive sections (120 μm apart) were then washed and mounted utilizing FluoroShield mounting medium with DAPI (Abcam). Double immuno-fluorescence was assessed using a Carl Zeiss LSM780 confocal scanning laser microscope with a 5 × air objective and 20 × or 40 × oil objective.

## Results

### Channelrhodopsin-2 (ChR2) Expression Patterns in Primary VC and Associated Cortico-cortical and Corticofugal Projections

Figure 1 and Supplementary Figure S1 show the expression patterns of the optogenetic construct, ChR2 in the primary VC (injection area) and its associated monosynaptic projection targets. CaMKIIα dependent ChR2(H134R) fused with mCherry was seen to primarily express in the infragranular layers of the primary VC and in the interlaminar projections from layer V pyramidal neurons to layer II/III in VC (Fig. 1a). The expression of ChR2 in the cell bodies of primary VC infragranular pyramidal/excitatory neurons as further confirmed (Fig. 1b), demonstrate that the injection location was centered in the infragranular layers.

ChR2 expression was also identified in the corticofugal projections from infragranular pyramidal neurons to the ipsilateral lateral geniculate nucleus (LGN) and superficial layers of the superior colliculus (SC) (Supplementary Figure S1a,b). This was expected since these regions are known to receive projections predominantly from layer V^68^ and layer VI^69^ of the primary VC. There was only minimal expression in the contralateral VC (Supplementary Figure S1c). This finding further indicates that the excitatory neurons in infragranular, not upper layers, were predominantly transfected. Otherwise, a strong expression in contralateral VC would be expected from the upper layers (e.g., layer II/III) as characterized by previous studies^70–72^. ChR2 expression was also found in the primary VC to lateral VC projections from layer V/VI primary VC neurons to layer VI in lateral VC (Fig. 1c). Additionally, ChR2 was expressed in the VC to AC projections that terminated at the infragranular layers of AC, albeit at a much lower density (Fig. 1d).

### Optogenetic Stimulation in VC Does Not Affect Baseline BOLD Activities in IC

**Figure 3:**
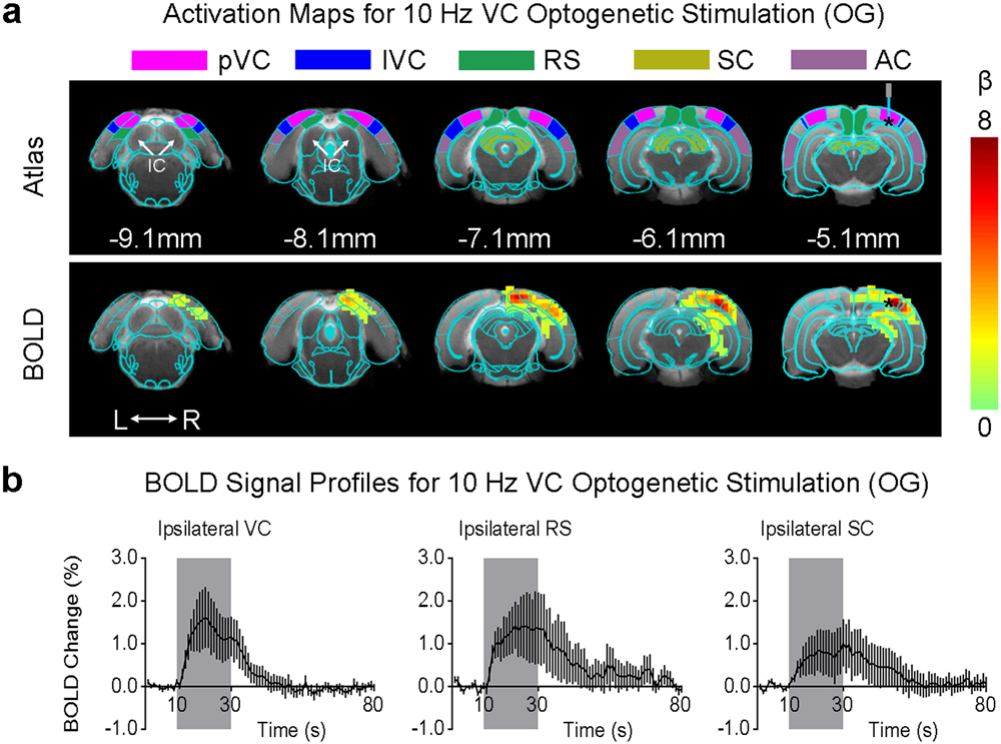
Optogenetic stimulation of the primary visual cortex at 10 Hz does not induce fMRI response in the IC. (**a**) Atlas (from the Paxinos & Watson) and activation (β) maps for the 10 Hz VC optogenetic stimulation (OG) overlaid on anatomical MRI. Activated voxels detected in the local VC, including pVC and lVC, RS, and ipsilateral SC and AC (n = 9; p < 0.05, corrected for FWE) are shown by the heat map. Abbreviations of atlas overlay are as follows: pVC (primary visual cortex), lVC (lateral visual cortex), RS (retrosplenial cortex), SC (superior colliculus), and AC (auditory cortex). (**b**) BOLD signal profiles in the significant voxels identified in (**a**). The results are presented as means ± standard error of the mean. Area in shade indicates the 20 s 10 Hz optogenetic stimulation.

During fMRI experiments, optogenetic stimulation targeted the infragranular layers of primary VC (Fig. 1a). Figure 3 presents the fMRI responses (p < 0.05, corrected for FWE) evoked by 10 Hz optogenetic stimulation only. As expected, strong BOLD responses were detected locally throughout the VC, including the primary and lateral regions. Meanwhile, responses were also observed in the ipsilateral retrosplenial cortex (RS), SC and hippocampus (HP), which participate in visuospatial navigation^73–75^. However, no responses were detected in the IC or other subcortical auditory nuclei in either hemisphere.

The fMRI responses to the optogenetic stimulation at other frequency were different from those to the 10 Hz stimulation. Supplementary Figure S2 shows that 1 Hz stimulation evoked significant responses (p < 0.05, corrected for FWE) in both brain hemispheres, including the ipsilateral VC and HP, contralateral VC, and bilateral AC. Similarly, no responses were detected in the subcortical auditory structures, including the IC. Meanwhile, as expected, no BOLD responses were present in naïve animals under the same light stimulation paradigms (10 Hz; Supplementary Figure S3).

### VC Optogenetic Stimulation Enhances Noise-Evoked fMRI Responses in IC

**Figure 4:**
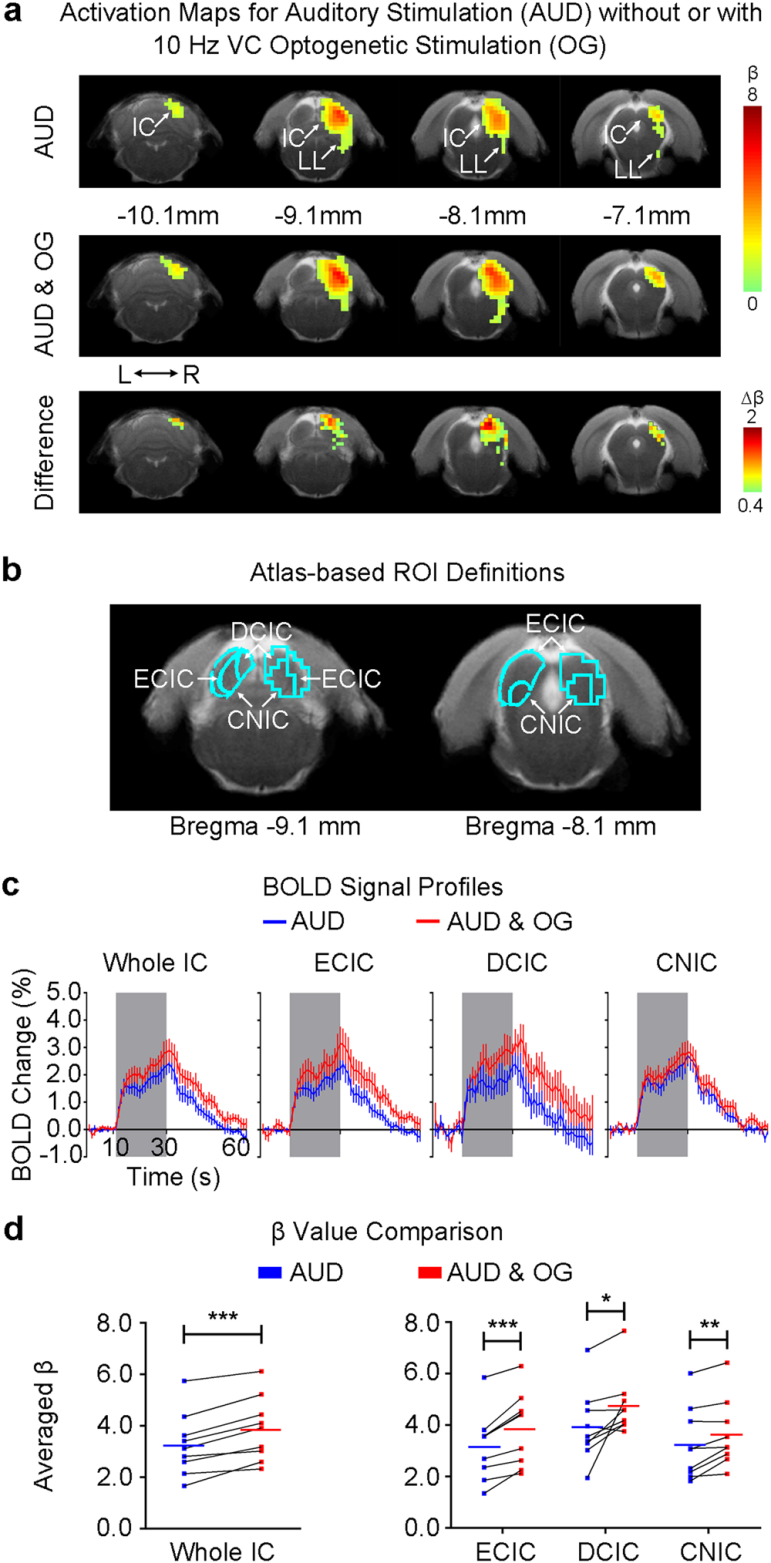
Optogenetic stimulation of the VC at 10 Hz enhances auditory fMRI response in the IC. (**a**) The activation (β) maps in the IC (and LL) for the auditory stimulation (AUD) without and with the 10 Hz VC optogenetic stimulation (OG), and the difference (Δβ) between two conditions. Activated voxels (n = 9; p < 0.05, corrected for FWE) are shown by the heat map, and in the difference map Δβ is further threshold at 0.4. VC activation generally increased the IC noise response. (**b**) Analysis ROIs defined in the external cortex of the IC (ECIC), dorsal cortex of the IC (DCIC) and the central nucleus of the IC (CNIC) (right side) as demarcated in Paxinos & Watson rat brain atlas (left side). The ECIC and CNIC ROIs contain voxels from both Bregma −9.1 mm and −8.1 mm, while the DCIC ROI from only Bregma −9.1 mm. (**c**) BOLD signal profiles extracted from the defined ROIs in the inferior colliculus (IC) and its subnuclei during the auditory stimulation (AUD; area in shade) without and with the 10 Hz VC optogenetic stimulation (OG). The results are presented as means ± standard error of the mean. (**d**) Comparison between the BOLD responses (β values) to the broadband noise stimulation without and with VC optogenetic stimulation in each ROI across individual animals. Note that the solid colored line (blue or red) in each plot represents the averaged β value. Optogenetic stimulation of the VC increased the IC responses, most prominently in the ECIC. Statistical comparisons were performed using paired two-sample t-test followed by Holm-Bonferroni correction with * for p < 0.05, **for p < 0.01, ***for p < 0.001 and n.s. for not significant.

Figure 4a shows the fMRI responses (p < 0.05, corrected for FWE) evoked by the broadband noise stimulation, prominently in the right (contralateral to auditory stimulation side) IC and lateral lemniscus (LL, a set of nuclei that project to the IC). The IC responses were significantly increased (p < 0.001, paired Student’s t test followed by Holm-Bonferroni correction, Fig. 4a,c) during 10 Hz optogenetic stimulation of the primary VC. This increase was prominent in the dorsal cortex and external cortex of the IC (DCIC: p < 0.05, paired Student’s t test followed by Holm-Bonferroni correction; ECIC: p < 0.001, paired Student’s t test followed by Holm-Bonferroni correction). The central nucleus of the IC (CNIC) response was moderately increased (CNIC: p < 0.01, paired Student’s t test followed by Holm-Bonferroni correction; Fig. 4b,d; also see the BOLD signal profiles during auditory stimulation in Fig. 4c). Although CNIC is known to only receive very few descending projections from AC^76^, it was still enhanced significantly by the 10 Hz VC optogenetic stimulation (Fig. 4). This occurred likely because the function of CNIC could be influenced by DCIC and ECIC via their reciprocal connections^77^.

In contrast, in a pilot experiment, noise-evoked IC responses were not altered during 1 Hz optogenetic stimulation in the VC (Supplementary Figure S4), suggesting the temporal specificity of optogenetic VC stimulation in enhancing IC auditory responses. No alteration was observed in IC responses to noise in naïve animals (10 Hz and 1 Hz; Supplementary Figures S5 and S6), confirming that the modulatory effects were caused by the optogenetic stimulation.

## Discussion

Combining optogenetics that enables cell-type specific neuromodulation and auditory fMRI that provides large view readout of auditory neural responses the present study offers a valuable approach to investigating cortical descending influences on auditory midbrain processing. The results show that VC optogenetic activation at 10 Hz facilitates auditory midbrain processing of broadband noise. The increased IC auditory responses do not arise from optogenetic stimulation alone. These findings indicate that the excitatory neurons in the infragranular layers of the VC play a critical role to drive cross-modal corticofugal modulatory effects in the auditory midbrain. Further, they suggest that cross-modal integration depends on the temporal patterns of neuronal activity within the VC, which can very well be necessary during multisensory processing in response to complex external stimulation.

Since its introduction, optogenetics has been widely used in all areas of neuroscience research^39,41,78,79^. While the vast majority of optogenetic studies are carried out with conventional electrophysiological methods, large-view imaging methods, particularly fMRI, have been gradually implemented to investigate the effects of optogenetic stimulation at brain-wide scale in recent years^46–53^. Currently, a number of studies indicated that optogenetic fMRI responses are dependent on the spatiotemporal patterns of the stimulation^49,50,52,53^. Therefore, the optogenetic stimulation paradigm in the current study was carefully designed and its spatiotemporal response characteristics were examined in detail prior to investigating its influences on auditory midbrain responses to external stimulation. Spatially, the optogenetic stimulation was targeted to the excitatory pyramidal neurons in the infragranular layers (i.e. layers V and VI) of the primary VC. In both auditory and visual systems, these neurons constitute the major source of corticofugal projections to the thalamus, midbrain and other lower levels of the respective pathway^54,55^. Temporal patterns of the optogenetic stimulation used in the present study were determined experimentally. The pulse duration and the intensity of the optogenetic stimulation were set in ranges that have been shown to induce robust BOLD fMRI responses in previous works^46,49,50,52,53^.

Our results showed that optogenetic activation of the VC does not evoke BOLD fMRI responses within IC or other auditory subcortical nuclei, though it induces responses in multiple cortical and subcortical regions distant from the stimulation site, particularly at low stimulation frequency (1 Hz). The exact mechanisms underlying such long-range responses remain to be further studied. Yet they are not entirely surprising given the existence of corticocortical projections, e.g. between the auditory and visual cortices^2,15,16^, corticofugal projections between VC and SC^68^, and corticohippocampal projections^80,81^. Moreover, we demonstrated the structural existence of these corticocortical and corticofugal projections (Fig. 1 and Supplementary Figure S1), further corroborating our observed evoked BOLD responses (Fig. 3).

It is worth noting that the detection of robust positive BOLD responses in fMRI experiments is dependent of the underlying electrical activity. It has been shown that evoked BOLD responses correlate the best with local field potentials (LFPs)^22,24^. LFPs reflect primarily a weighted average of the dendritic components of the synaptic signals of a neural population (i.e., the sum of excitatory and inhibitory electrical activity at the synapse). Taken together, to detect a robust BOLD response at a certain region, an appreciable summation of evoked synaptic potentials is required. Corticocollicular projections terminate mainly on distal dendritic profiles of IC neurons and not their cell bodies^82^, suggesting that these projections are modulatory and non-driving. As BOLD responses are primarily driven by the sum of electrical activity caused by excitation, it is not surprising that we were unable to detect evoked BOLD responses in IC upon the optogenetic activation of VC alone. Although previous electrophysiological recordings showed that visual stimuli alone can evoke spiking responses in IC neurons^35,36^, it is important to distinguish the stimulation protocols used. Previous studies presented visual stimuli to the eye, whereby it is difficult to determine the large-scale influences of VC alone (i.e., driving or modulatory) on IC response due to the unspecific stimulus^83^. Hence, inputs from VC to IC may be primarily modulatory given the specificity afforded by the optogenetic stimulation.

Our results showed that optogenetic activation of the VC enhances IC auditory responses to noise stimulation. This corroborates our recent ablation results that found the decreased IC noise responses after bilateral ablation of the VC^25^. Together, they indicate that the VC normally facilitates basic sound processing in the auditory midbrain. The increase of noise responses in the present study was most prominent in dorsal cortex of the IC (DCIC) and external cortex of the IC (ECIC), whereas in our previous ablation study it was most prominent in the central nucleus of the IC (CNIC) but not in both DCIC and ECIC. Such differences are likely explained by the distinct properties of the cortical manipulations employed by the two studies. Compared to cortical ablation, focal optogenetic stimulation involves a very local and precise region (the infragranular primary VC) and can initiate spatiotemporally structured excitatory signals that can eventually lead to dynamic yet stable excitation-inhibition interactions. Moreover, such precision afforded by optogenetic stimulation enables the characterization of distinct, heterogeneous influences on individual IC subnuclei. Furthermore, optogenetic stimulation is less invasive, whereas the long recovery time used in the ablation study may permit neural plasticity mechanisms to promote differential changes in IC subnuclei response properties.

The exact mechanism(s) remains to be elucidated in the future studies. There can be several neural pathway(s) that likely underlie the large-scale modulatory influences from VC on auditory midbrain processing revealed by our results. Firstly, feedback to the IC can be relayed through direct projections from the lateral VC^12–14^. However, previous studies only present evidence for direct anatomical projections. Currently, the exact functional influence of these projections remains unknown. Secondly, such feedback can also be relayed to the IC through the auditory cortex (AC). For example, previous studies indicated that AC neurons could be modulated by sub-threshold responses induced by the visual inputs. Modulatory effects on IC neurons have been proposed to be mediated through decreased/increased inhibitory/excitatory inputs from the VC^5^ and AC^17,76^, and/or changes in the membrane potential of neurons^28^. Such modulations will affect the excitability of IC neurons, thereby changing their auditory response properties. Other areas may also relay visual feedback to the IC too, such as the auditory or visual thalamus (which are also targets of corticofugal projections), the retrosplenial cortex (RS), or the superior colliculus (SC, which is known for mediating multisensory responses). Meanwhile, given the hippocampal-IC interactions^84–86^, the hippocampus could also mediate the VC cross-modal influences, since it exhibited responsivity during 10 Hz optogenetic stimulation. Future studies will elucidate these underlying circuitries and mechanisms through electrophysiological recordings.

In the present study, auditory fMRI responses to the broadband noise stimulation were also observed in the right medial geniculate body (MGB, p < 0.05 but not significant after correction for FWE) and lateral lemniscus (LL, p < 0.05, corrected for FWE). During optogenetic stimulation of the VC, the MGB response exhibited an increasing trend (p < 0.05 for paired Student’s t test but does not pass Holm-Bonferroni correction; Supplementary Figure S7), suggesting a possibility that VC co-modulates auditory processing at multiple stages immediately prior to the cortex. The LL responses were not significantly influenced by the optogenetic stimulation (Supplementary Figure S7). Nevertheless, as our imaging protocols were optimized to detect differences in IC responses and not for these structures, future studies are needed to enhance our understanding of the corticofugal influences on these structures. Our fMRI protocols were optimal for IC because the MRI surface coil was centered on and MRI shimming was restricted to imaging slices covering the IC. Together, they ensure that the magnetic field homogeneity^87^ within a restricted region in the vicinity of the IC is optimized but at the detriment to regions that are far away from IC, such as MGB and AC.

In the present study, the optogenetic stimulation was targeted to the infragranular layers of the VC. Accuracy in both ChR2 injection and fiber implantation steps was critical for achieving precise stimulation. Our histology results confirmed that ChR2 was expressed successfully primarily in the deep layers of the VC. Expression in the superficial layers was limited (Fig. 1a). Meanwhile, anatomical MRI confirmed that the fibre tip was at the right spot. As blue light is heavily scattered in the brain tissue, with less than 10% light power at 500 μm from tip^41,88^, neurons outside the infragranular layers should not have been effectively stimulated.

Besides the issue of spatial specificity in optogenetic targeting and stimulation, the temporal characteristics of stimulation also determine the responses. Recent studies demonstrated that optogenetically evoked BOLD fMRI responses are highly dependent on the spatiotemporal patterns of the stimulation^50,52,53,89^. Our choice of 10 Hz and 1 Hz stimulation to excite the VC was partly motivated by the findings from us and others^53,90^ that demonstrate that 10 Hz activity is predominant in the infragranular excitatory pyramidal neurons of the VC^90^, while 1 Hz thalamic optogenetic stimulation has the propensity to induce polysynaptic and widespread visual cortical neural activity^53^. However, these findings are only indicative of the complex feedforward and feedback interactions that may occur across different layers within the primary VC. The exact neural activity patterns initiated by these interactions can differ in temporal characteristic from the optogenetic stimulus. Therefore, the key question remains what constitutes a physiologically relevant stimulus in the VC. Further, what and how specific neural activity spatiotemporal patterns initiated in the VC mediate cross-modal corticofugal modulations.

The present study represents a novel effort to combine optogenetic stimulation with an external sensory stimulation in an fMRI study. The bi-modal stimulation was presented to different brain hemispheres in this study (auditory to the left side and optogenetic to the right side). In other words, this study investigated the VC descending influences on the ipsilateral auditory midbrain, as auditory fMRI responses were mainly observed in the right hemisphere. This design assumed that brain corticofugal projections primarily target ipsilateral nuclei^8–13^. Corticofugal input is also expected to modulate the contralateral auditory midbrain^17^, which can be investigated in future studies. During the bi-modal stimulation, the optogenetic stimulation was presented from 10 s before to 10 s after the broadband noise stimulation. Such a design allowed auditory fMRI responses to evolve while the optogenetic modulation effects remained stable. In cases where the optogenetic stimulation directly evokes responses in the IC, this paradigm may provide flexibility to account for such responses during data analysis. Nevertheless, it is imperative to investigate how corticofugal influences on auditory midbrain responses depend on the relative timing between the bi-modal stimulation in future studies (e.g. the optogenetic and auditory stimulation are turned on and off simultaneously, etc.).

The present study demonstrates the feasibility of combining optogenetics with auditory fMRI to investigate cortical descending modulation of auditory midbrain processing. Several lines of experiments are stimulated by our present results. First, investigating other cortical optogenetic stimulation strategies will further reveal how visual and non-visual cortical regions modulate auditory midbrain processing. For example, future studies may employ optogenetic stimulation that mimics the neuronal activation patterns in the cortex during auditory processing (e.g. in closed-loop form^91^) or complementarily optogenetic silencing strategies^92^ to elucidate the network activity during corticofugal modulation. Second, systematically evaluating cortical (including primary/lateral VC, AC or other cortices) influences during other auditory midbrain processing paradigms assessed by BOLD fMRI^56,61,62,64,66^ may further advance our understanding of the corticofugal modulation process across and within sensory modalities. Third, examining the functional integrity of the auditory descending pathways in hearing disorders, such as hearing loss^93^ or tinnitus^30^, and changes during drug interventions^94^ may provide insights for the design of brain stimulation strategies or prosthetics as therapeutics of hearing disorders^95,96^.

## Conclusion

This study demonstrates the promise of combining optogenetics with auditory fMRI for investigation of visual cortical descending influences on auditory midbrain responses. Optogenetic activation of predominantly excitatory pyramidal neurons in the infragranular layers of primary visual cortex does not directly evoke BOLD responses in the auditory midbrain, but enhances the auditory midbrain responses to broadband noise stimulation. The results indicate that the visual cortex facilitates auditory midbrain processing of basic sound features, and such effect is likely driven by excitatory neurons in the infragranular layers. Applications of this optogenetic auditory fMRI approach can guide detailed electrophysiological studies in the future.

## Electronic supplementary material

Supplementary Information

## References

[1] Winer JA (2006). Decoding the auditory corticofugal systems. Hear Res.

[2] Falchier A, Clavagnier S, Barone P, Kennedy H (2002). Anatomical evidence of multimodal integration in primate striate cortex. J Neurosci.

[3] Wallace MT, Ramachandran R, Stein BE (2004). A revised view of sensory cortical parcellation. Proc Natl Acad Sci USA.

[4] Mowery TM, Kotak VC, Sanes DH (2016). The onset of visual experience gates auditory cortex critical periods. Nat Commun.

[5] Bizley JK, King AJ (2009). Visual influences on ferret auditory cortex. Hear Res.

[6] Meredith MA, Allman BL, Keniston LP, Clemo HR (2009). Auditory influences on non-auditory cortices. Hear Res.

[7] Malmierca MS (2003). The structure and physiology of the rat auditory system: an overview. Int Rev Neurobiol.

[8] Bajo VM, Moore DR (2005). Descending projections from the auditory cortex to the inferior colliculus in the gerbil, Meriones unguiculatus. J Comp Neurol.

[9] Bajo VM, Nodal FR, Bizley JK, Moore DR, King AJ (2007). The ferret auditory cortex: descending projections to the inferior colliculus. Cereb Cortex.

[10] Coomes DL, Schofield RM, Schofield BR (2005). Unilateral and bilateral projections from cortical cells to the inferior colliculus in guinea pigs. Brain Res.

[11] Schofield BR (2009). Projections to the inferior colliculus from layer VI cells of auditory cortex. Neuroscience.

[12] Cooper MH, Young PA (1976). Cortical projections to the inferior colliculus of the cat. Exp Neurol.

[13] Dong, H.W. *The Allen reference atlas: A digital color brain atlas of the C57Bl/6J male mouse*, (John Wiley & Sons Inc, 2008).

[14] Oh SW (2014). A mesoscale connectome of the mouse brain. Nature.

[15] Budinger E, Heil P, Hess A, Scheich H (2006). Multisensory processing via early cortical stages: Connections of the primary auditory cortical field with other sensory systems. Neuroscience.

[16] Campi KL, Bales KL, Grunewald R, Krubitzer L (2010). Connections of auditory and visual cortex in the prairie vole (Microtus ochrogaster): evidence for multisensory processing in primary sensory areas. Cereb Cortex.

[17] Bajo VM, King AJ (2012). Cortical modulation of auditory processing in the midbrain. Front Neural Circuits.

[18] Stebbings KA, Lesicko AM, Llano DA (2014). The auditory corticocollicular system: molecular and circuit-level considerations. Hear Res.

[19] Finney EM, Fine I, Dobkins KR (2001). Visual stimuli activate auditory cortex in the deaf. Nat Neurosci.

[20] Lomber SG, Meredith MA, Kral A (2010). Cross-modal plasticity in specific auditory cortices underlies visual compensations in the deaf. Nat Neurosci.

[21] Petrus E (2014). Crossmodal induction of thalamocortical potentiation leads to enhanced information processing in the auditory cortex. Neuron.

[22] Logothetis NK, Pauls J, Augath M, Trinath T, Oeltermann A (2001). Neurophysiological investigation of the basis of the fMRI signal. Nature.

[23] Ogawa S, Lee TM, Kay AR, Tank DW (1990). Brain magnetic resonance imaging with contrast dependent on blood oxygenation. Proc Natl Acad Sci USA.

[24] Logothetis NK, Wandell BA (2004). Interpreting the BOLD signal. Annu Rev Physiol.

[25] Gao PP, Zhang JW, Fan SJ, Sanes DH, Wu EX (2015). Auditory midbrain processing is differentially modulated by auditory and visual cortices: An auditory fMRI study. Neuroimage.

[26] Yan W, Suga N (1998). Corticofugal modulation of the midbrain frequency map in the bat auditory system. Nat Neurosci.

[27] Ma X, Suga N (2001). Corticofugal modulation of duration-tuned neurons in the midbrain auditory nucleus in bats. Proc Natl Acad Sci USA.

[28] Yan J, Ehret G (2002). Corticofugal modulation of midbrain sound processing in the house mouse. Eur J Neurosci.

[29] Yan J, Zhang Y, Ehret G (2005). Corticofugal shaping of frequency tuning curves in the central nucleus of the inferior colliculus of mice. J Neurophysiol.

[30] Zhang Y, Suga N, Yan J (1997). Corticofugal modulation of frequency processing in bat auditory system. Nature.

[31] Nakamoto KT, Jones SJ, Palmer AR (2008). Descending projections from auditory cortex modulate sensitivity in the midbrain to cues for spatial position. J Neurophysiol.

[32] Popelar J (2016). Cooling of the auditory cortex modifies neuronal activity in the inferior colliculus in rats. Hear Res.

[33] Syka J, Radil-Weiss T (1973). Acoustical responses of inferior colliculus neurons in rats influenced by sciatic nerve stimulation and light flashes. The International journal of neuroscience.

[34] Tawil RN, Saade NE, Bitar M, Jabbur SJ (1983). Polysensory interactions on single neurons of cat inferior colliculus. Brain Res.

[35] Mascetti GG, Strozzi L (1988). Visual cells in the inferior colliculus of the cat. Brain Res.

[36] Porter KK, Metzger RR, Groh JM (2007). Visual- and saccade-related signals in the primate inferior colliculus. Proc Natl Acad Sci USA.

[37] Bulkin DA, Groh JM (2012). Distribution of visual and saccade related information in the monkey inferior colliculus. Front Neural Circuits.

[38] Bajo VM, Nodal FR, Moore DR, King AJ (2010). The descending corticocollicular pathway mediates learning-induced auditory plasticity. Nat Neurosci.

[39] Xiong XR (2015). Auditory cortex controls sound-driven innate defense behaviour through corticofugal projections to inferior colliculus. Nat Commun.

[40] Boyden ES, Zhang F, Bamberg E, Nagel G, Deisseroth K (2005). Millisecond-timescale, genetically targeted optical control of neural activity. Nat Neurosci.

[41] Zhang F (2010). Optogenetic interrogation of neural circuits: technology for probing mammalian brain structures. Nature protocols.

[42] Hausser M (2014). Optogenetics: the age of light. Nature methods.

[43] Mattis J (2012). Principles for applying optogenetic tools derived from direct comparative analysis of microbial opsins. Nature methods.

[44] Deisseroth K (2015). Optogenetics: 10 years of microbial opsins in neuroscience. Nat Neurosci.

[45] Warden MR, Cardin JA, Deisseroth K (2014). Optical neural interfaces. Annu Rev Biomed Eng.

[46] Lee JH (2010). Global and local fMRI signals driven by neurons defined optogenetically by type and wiring. Nature.

[47] Yu, X., *et al*. Sensory and optogenetically driven single-vessel fMRI. *Nat Methods* (2016). 10.1038/nmeth.3765 PMC6298439 26855362

[48] Albers F, Schmid F, Wachsmuth L, Faber C (2018). Line scanning fMRI reveals earlier onset of optogenetically evoked BOLD response in rat somatosensory cortex as compared to sensory stimulation. Neuroimage.

[49] Weitz AJ (2015). Optogenetic fMRI reveals distinct, frequency-dependent networks recruited by dorsal and intermediate hippocampus stimulations. Neuroimage.

[50] Liu J (2015). Frequency-selective control of cortical and subcortical networks by central thalamus. eLife.

[51] Ferenczi EA (2016). Prefrontal cortical regulation of brainwide circuit dynamics and reward-related behavior. Science.

[52] Chan RW (2017). Low-frequency hippocampal-cortical activity drives brain-wide resting-state functional MRI connectivity. Proc Natl Acad Sci USA.

[53] Leong AT (2016). Long-range projections coordinate distributed brain-wide neural activity with a specific spatiotemporal profile. Proc Natl Acad Sci USA.

[54] Longstaff, A. Instant notes in neuroscience (BIOS instant notes). (Abingdon: Taylor & Francis, 2005).

[55] Custo Greig LF, Woodworth MB, Galazo MJ, Padmanabhan H, Macklis JD (2013). Molecular logic of neocortical projection neuron specification, development and diversity. Nat Rev Neurosci.

[56] Gao PP, Zhang JW, Cheng JS, Zhou IY, Wu EX (2014). The inferior colliculus is involved in deviant sound detection as revealed by BOLD fMRI. Neuroimage.

[57] Gao PP, Zhang JW, Chan RW, Leong AT, Wu EX (2015). BOLD fMRI study of ultrahigh frequency encoding in the inferior colliculus. Neuroimage.

[58] Lau C, Zhang JW, McPherson B, Pienkowski M, Wu EX (2015). Long-term, passive exposure to non-traumatic acoustic noise induces neural adaptation in the adult rat medial geniculate body and auditory cortex. Neuroimage.

[59] Lau C, Pienkowski M, Zhang JW, McPherson B, Wu EX (2015). Chronic exposure to broadband noise at moderate sound pressure levels spatially shifts tone-evoked responses in the rat auditory midbrain. Neuroimage.

[60] Chan KC, Xing KK, Cheung MM, Zhou IY, Wu EX (2010). Functional MRI of postnatal visual development in normal and hypoxic-ischemic-injured superior colliculi. Neuroimage.

[61] Cheung MM (2012). BOLD fMRI investigation of the rat auditory pathway and tonotopic organization. Neuroimage.

[62] Cheung MM (2012). High fidelity tonotopic mapping using swept source functional magnetic resonance imaging. Neuroimage.

[63] Lau C (2013). Noninvasive fMRI investigation of interaural level difference processing in the rat auditory subcortex. PLoS One.

[64] Zhang JW (2013). Functional magnetic resonance imaging of sound pressure level encoding in the rat central auditory system. Neuroimage.

[65] Zhou IY (2014). Brain resting-state functional MRI connectivity: morphological foundation and plasticity. Neuroimage.

[66] Zhang, J. W. *et al*. bSSFP fMRI study of sound amplitude modulation in inferior colliculus. In *Proceedings of the 21st Annual Meeting of ISMRM*, *Salt Lake City*, *USA* 302 (2013).

[67] Chan RW (2015). Structural and Functional Brain Remodeling during Pregnancy with Diffusion Tensor MRI and Resting-State Functional MRI. PLoS One.

[68] Kim EJ, Juavinett AL, Kyubwa EM, Jacobs MW, Callaway EM (2015). Three Types of Cortical Layer 5 Neurons That Differ in Brain-wide Connectivity and Function. Neuron.

[69] Briggs F (2010). Organizing Principles of Cortical Layer 6. Front Neural Circuits.

[70] Fame RM, MacDonald JL, Macklis JD (2011). Development, specification, and diversity of callosal projection neurons. Trends Neurosci.

[71] Petreanu L, Huber D, Sobczyk A, Svoboda K (2007). Channelrhodopsin-2-assisted circuit mapping of long-range callosal projections. Nat Neurosci.

[72] Zhou J (2013). Axon position within the corpus callosum determines contralateral cortical projection. Proc Natl Acad Sci USA.

[73] Howard LR (2014). The hippocampus and entorhinal cortex encode the path and Euclidean distances to goals during navigation. Curr Biol.

[74] Czajkowski R (2014). Encoding and storage of spatial information in the retrosplenial cortex. Proc Natl Acad Sci USA.

[75] Dash S, Yan X, Wang H, Crawford JD (2015). Continuous updating of visuospatial memory in superior colliculus during slow eye movements. Curr Biol.

[76] Malmierca MS, Anderson LA, Antunes FM (2015). The cortical modulation of stimulus-specific adaptation in the auditory midbrain and thalamus: a potential neuronal correlate for predictive coding. Frontiers in systems neuroscience.

[77] Malmierca MS, Merchán MA (2004). Auditory system. The rat nervous system.

[78] Beltramo R (2013). Layer-specific excitatory circuits differentially control recurrent network dynamics in the neocortex. Nat Neurosci.

[79] Kim CK, Adhikari A, Deisseroth K (2017). Integration of optogenetics with complementary methodologies in systems neuroscience. Nat Rev Neurosci.

[80] Ji D, Wilson MA (2007). Coordinated memory replay in the visual cortex and hippocampus during sleep. Nat Neurosci.

[81] Lavenex P, Amaral DG (2000). Hippocampal-neocortical interaction: a hierarchy of associativity. Hippocampus.

[82] Saldana E, Feliciano M, Mugnaini E (1996). Distribution of descending projections from primary auditory neocortex to inferior colliculus mimics the topography of intracollicular projections. J Comp Neurol.

[83] Gruters KG, Groh JM (2012). Sounds and beyond: multisensory and other non-auditory signals in the inferior colliculus. Front Neural Circuits.

[84] Pedemonte M, Pena JL, Velluti RA (1996). Firing of inferior colliculus auditory neurons is phase-locked to the hippocampus theta rhythm during paradoxical sleep and waking. Exp Brain Res.

[85] Kraus KS, Canlon B (2012). Neuronal connectivity and interactions between the auditory and limbic systems. Effects of noise and tinnitus. Hear Res.

[86] Liberman T, Velluti RA, Pedemonte M (2009). Temporal correlation between auditory neurons and the hippocampal theta rhythm induced by novel stimulations in awake guinea pigs. Brain Res.

[87] Balteau E, Hutton C, Weiskopf N (2010). Improved shimming for fMRI specifically optimizing the local BOLD sensitivity. Neuroimage.

[88] Yizhar O, Fenno LE, Davidson TJ, Mogri M, Deisseroth K (2011). Optogenetics in neural systems. Neuron.

[89] Lee HJ (2016). Activation of Direct and Indirect Pathway Medium Spiny Neurons Drives Distinct Brain-wide Responses. Neuron.

[90] Sun W, Dan Y (2009). Layer-specific network oscillation and spatiotemporal receptive field in the visual cortex. Proc Natl Acad Sci USA.

[91] Grosenick L, Marshel JH, Deisseroth K (2015). Closed-loop and activity-guided optogenetic control. Neuron.

[92] Raimondo JV, Kay L, Ellender TJ, Akerman CJ (2012). Optogenetic silencing strategies differ in their effects on inhibitory synaptic transmission. Nat Neurosci.

[93] Geleoc GS, Holt JR (2014). Sound strategies for hearing restoration. Science.

[94] Aston-Jones G, Deisseroth K (2013). Recent advances in optogenetics and pharmacogenetics. Brain Res.

[95] Jeschke M, Moser T (2015). Considering optogenetic stimulation for cochlear implants. Hear Res.

[96] Moser T (2015). Optogenetic stimulation of the auditory pathway for research and future prosthetics. Current opinion in neurobiology.

