# Supplementary material for: Optogenetic auditory fMRI reveals the effects of visual cortical inputs on auditory midbrain response
Source: Sci Rep. 2018 Jun 7;8:8736. doi: 10.1038/s41598-018-26568-1 (PMC5992211; doi:10.1038/s41598-018-26568-1)
Supplement: Supplementary file 1 — Supplementary Information [file 41598_2018_26568_MOESM1_ESM.doc]

Supplementary Materials

Optogenetic auditory fMRI reveals the effects of visual cortical inputs on auditory midbrain response

Alex T. L. Leong ^a, b, 1^, Celia M. Dong ^a, b^, Patrick P. Gao ^a, b, 1^, Russell W. Chan ^a, b, 1^,
Anthea To ^a, b^, Dan H. Sanes ^c^ and Ed X. Wu ^a, b, d, e,*^

^a^Laboratory of Biomedical Imaging and Signal Processing, ^b^Department of Electrical and Electronic Engineering, The University of Hong Kong, Pokfulam, Hong Kong SAR, China, ^c^Center for Neural Science, New York University, New York, NY 10003, United States, ^d^School of Biomedical Sciences and ^e^Department of Medicine, Li Ka Shing Faculty of Medicine, The University of Hong Kong, Pokfulam, Hong Kong SAR, China.

^1^These authors contributed equally to this work.

^*^Correspondence should be addressed to Ed X. Wu, PhD: Laboratory of Biomedical Imaging and Signal Processing, Department of Electrical and Electronic Engineering, School of Biomedical Sciences and Department of Medicine, The University of Hong Kong, Pokfulam, Hong Kong SAR, China. Fax: +852-2859-8738. Tel: +852-2859-7096. Email: [ewu@eee.hku.hk](mailto:ewu@eee.hku.hk)


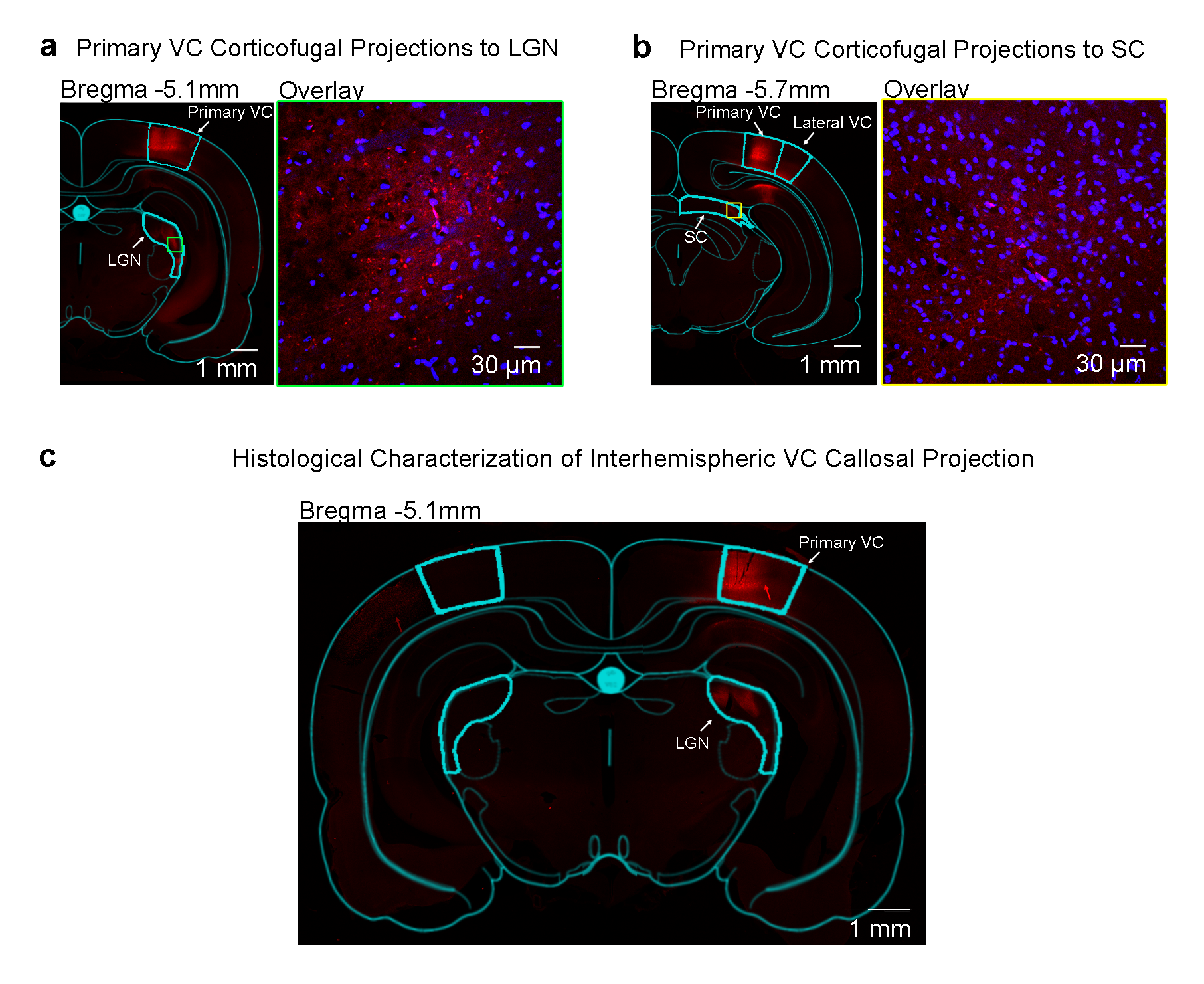


**Supplementary Figure S1 Corticofugal projections from the primary visual cortex (pVC) to the visual thalamus, lateral geniculate nucleus (LGN) and superior colliculus (SC)** (**a**) Left: Low-magnification confocal images showing the ChR2 expression in pVC and the corticofugal projections to LGN (emboldened blue boundary). Right: High-magnification confocal image in the LGN showing no colocalization between mCherry (red) and DAPI (blue), demonstrating monosynaptic corticofugal projections from infragranular pVC neurons to LGN neurons. (**b**) Left: Low-magnification confocal images showing the ChR2 expression in pVC and the corticofugal projections to SC (emboldened blue boundary). Right: High-magnification confocal image in the SC showing no colocalization between mCherry and DAPI, demonstrating monosynaptic corticofugal projections from infragranular pVC neurons to SC neurons. (**c**) Low-magnification confocal image showing the ChR2 expression in both hemispheres, namely the ipsilateral primary VC, contralateral VC and ipsilateral LGN. Note that there is minimal expression in contralateral VC (dark red arrow) when compared to the ipsilateral VC (bright red arrow).

**
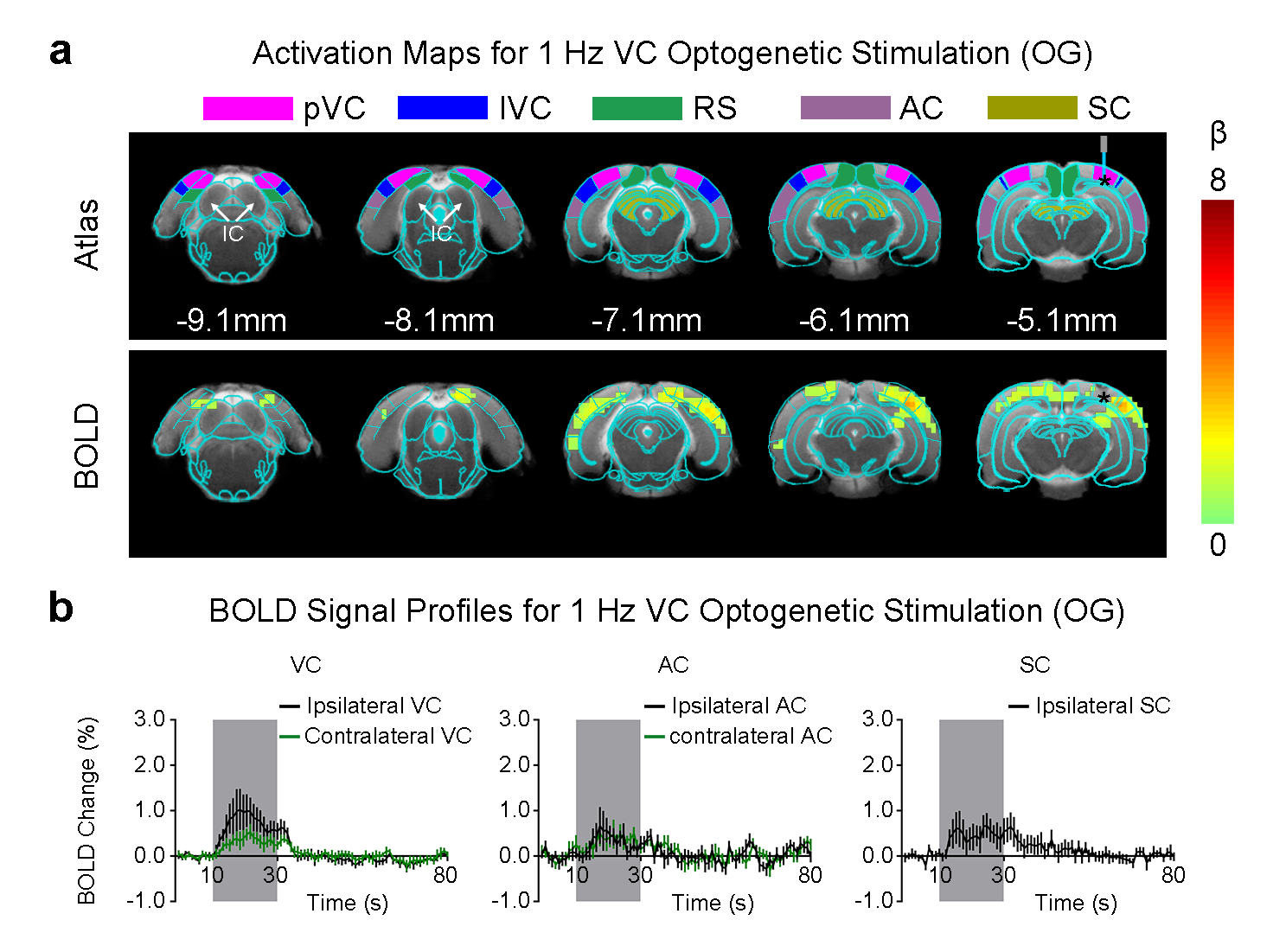
**

**Supplementary Figure S2 BOLD fMRI responses to 1 Hz optogenetic stimulation of the VC** (**a**) 1 Hz VC optogenetic stimulation (OG) evokes BOLD responses (n = 9; p<0.05, corrected for FWE) in both brain hemispheres, including the ipsilateral VC and SC, contralateral VC and bilateral AC. Abbreviations of Paxinos atlas overlay are as follows: pVC (primary visual cortex), lVC (lateral visual cortex), RS (retrosplenial cortex), AC (auditory cortex), and SC (superior colliculus). (**b**) BOLD signal profiles in the significant voxels identified in (a). The results are presented as means ± standard error of the mean. Area in shade indicates the 20 s 1 Hz optogenetic stimulation.


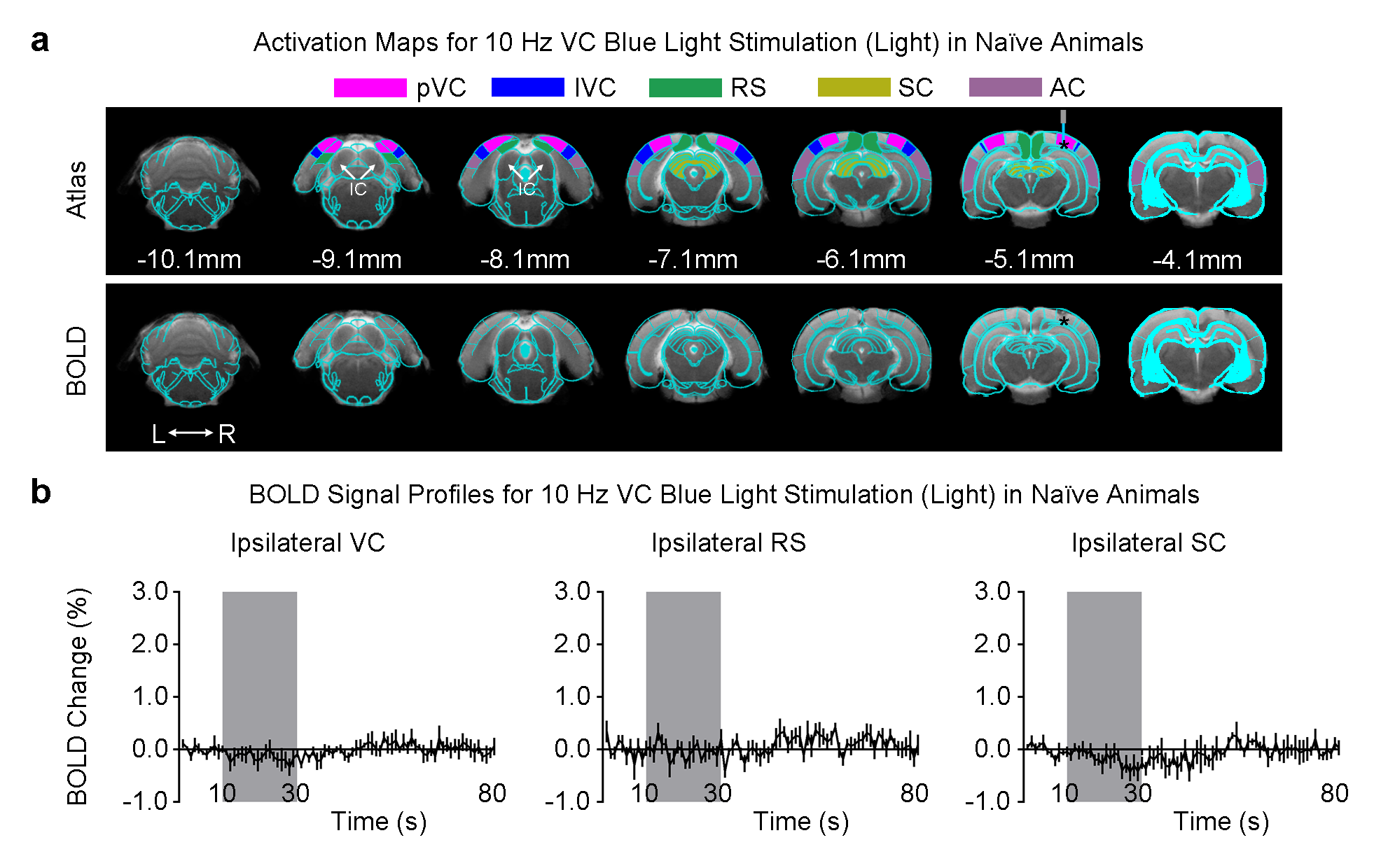


**Supplementary Figure S3 Absence of BOLD fMRI responses to 10 Hz blue light stimulation of the VC in naïve animals** (**a**) 10 Hz VC blue light stimulation (Light) in naïve animals do not evoke BOLD responses (n = 5; p<0.05, corrected for FWE) in visual regions, including the VC, RS and SC. Abbreviations of Paxinos atlas overlay are as follows: pVC (primary visual cortex), lVC (lateral visual cortex), RS (retrosplenial cortex), SC (superior colliculus), and AC (auditory cortex). (**b**) BOLD signal profiles extracted from significant voxels that are defined from activated regions in Fig. 3a (i.e., ipsilateral VC, RS and SC). The results are presented as means ± standard error of the mean. Area in shade indicates the 20 s 10 Hz blue light stimulation.


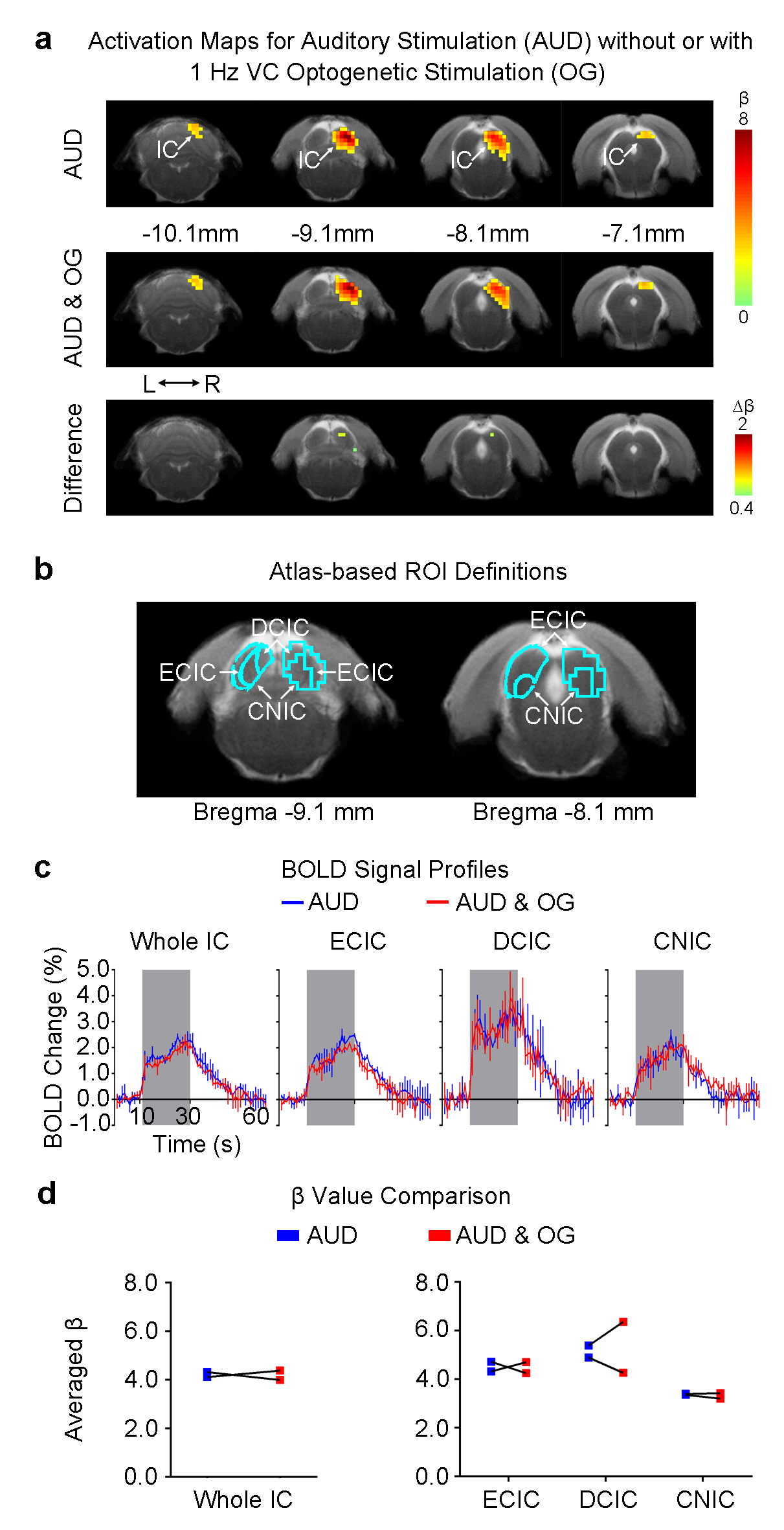


**Supplementary Figure S4 Optogenetic stimulation of the VC at 1 Hz minimally affects auditory fMRI response in the IC.** (**a**) The activation (β) maps in the IC for the auditory stimulation (AUD) without and with the 1 Hz VC optogenetic stimulation (OG), and the difference (Δβ) between two conditions. Activated voxels (n = 2; p<0.05, corrected for FWE) are shown by the heat map, and in the difference map Δβ is further threshold at 0.4. VC activation generally increased the IC noise response. (**b**) Analysis ROIs defined in the external cortex of the IC (ECIC), dorsal cortex of the IC (DCIC) and the central nucleus of the IC (CNIC) (right side) as demarcated in Paxinos & Watson rat brain atlas (left side). The ECIC and CNIC ROIs contain voxels from both Bregma -9.1 mm and -8.1 mm, while the DCIC ROI from only Bregma -9.1 mm. (**c**) BOLD signal profiles extracted from the defined ROIs in the inferior colliculus (IC) and its subnuclei during the auditory stimulation (AUD; area in shade) without and with the 1 Hz VC optogenetic stimulation (OG). (**d**) Comparison between the BOLD responses (β values) to the broadband noise stimulation without and with VC optogenetic stimulation in each ROI across individual animals. Optogenetic stimulation of the VC at 1 Hz minimally affects IC responses.


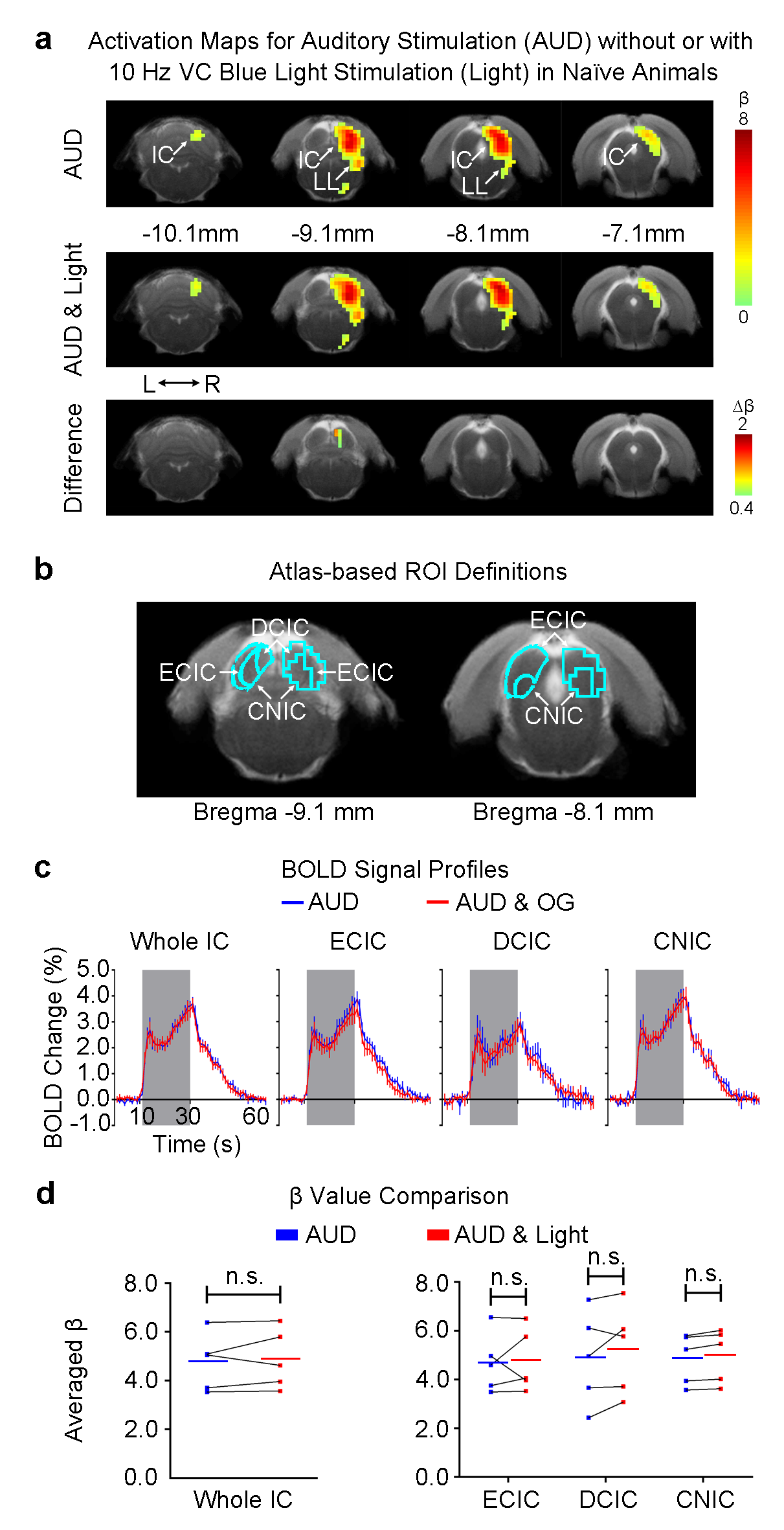


**Supplementary Figure S5 Blue light stimulation of the VC at 10 Hz does not alter auditory fMRI response in the IC of naïve animals.** (**a**) The activation (β) maps in the IC (and LL) for the auditory stimulation (AUD) without and with the 10 Hz VC blue light stimulation (Light), and the difference (Δβ) between two conditions. Activated voxels (n = 5; p<0.05, corrected for FWE) are shown by the heat map, and in the difference map Δβ is further threshold at 0.4. VC activation generally increased the IC noise response. (**b**) Analysis ROIs defined in the external cortex of the IC (ECIC), dorsal cortex of the IC (DCIC) and the central nucleus of the IC (CNIC) (right side) as demarcated in Paxinos & Watson rat brain atlas (left side). The ECIC and CNIC ROIs contain voxels from both Bregma -9.1 mm and -8.1 mm, while the DCIC ROI from only Bregma -9.1 mm. (**c**) BOLD signal profiles extracted from the defined ROIs in the inferior colliculus (IC) and its subnuclei during the auditory stimulation (AUD; area in shade) without and with the 10 Hz VC blue light stimulation (Light). (**d**) Comparison between the BOLD responses (β values) to the broadband noise stimulation without and with VC blue light stimulation in each ROI across individual animals. Note that the solid colored line (blue or red) in each plot represents the averaged β value. No significant differences in IC noise response were observed. Statistical comparisons were performed using paired two-sample t-test followed by Holm-Bonferroni correction with n.s. for not significant.


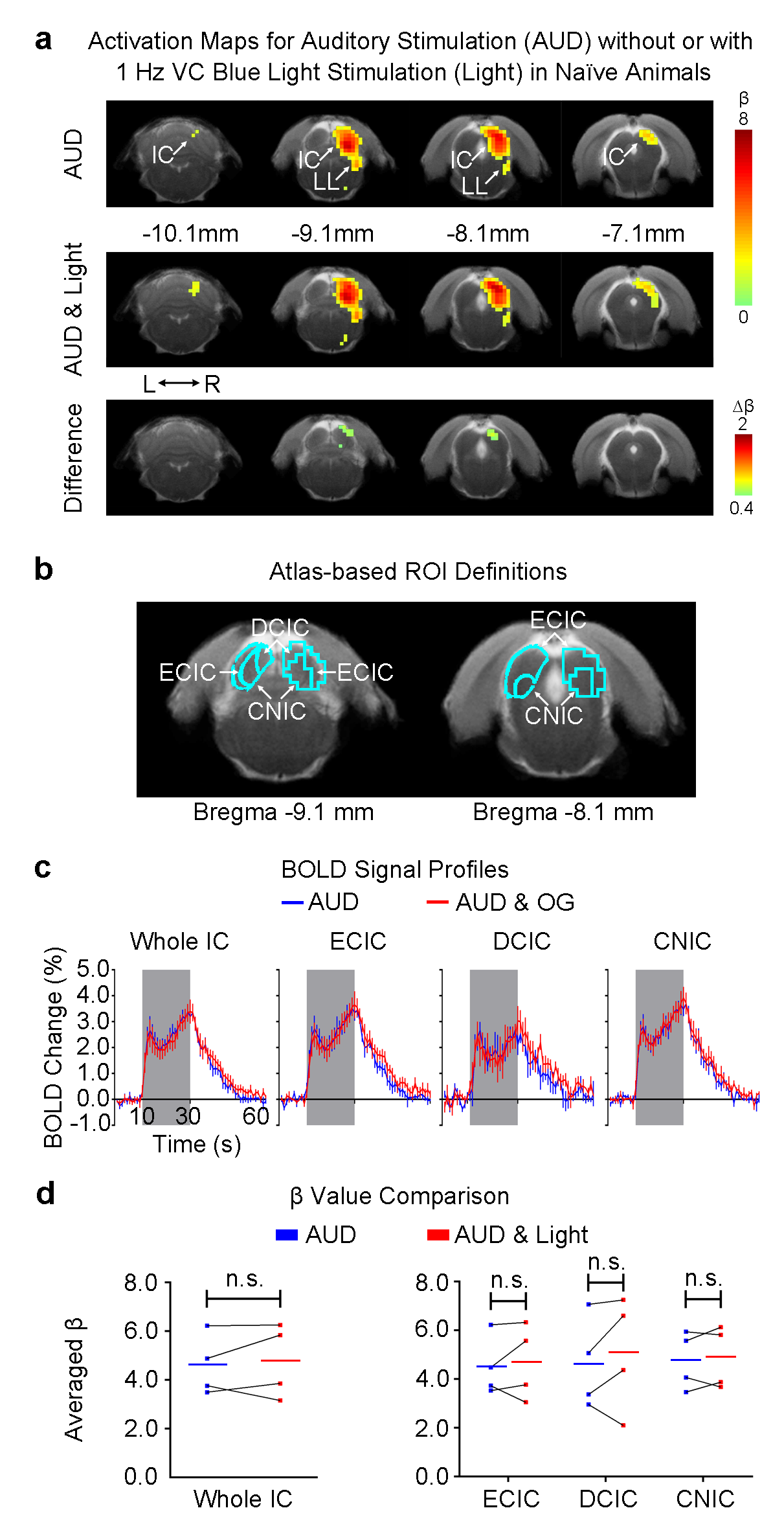


**Supplementary Figure S6 Blue light stimulation of the VC at 1 Hz also does not alter auditory fMRI response in the IC of naïve animals.** (**a**) The activation (β) maps in the IC (and LL) for the auditory stimulation (AUD) without and with the 1 Hz VC blue light stimulation (Light), and the difference (Δβ) between two conditions. Activated voxels (n = 5; p<0.05, corrected for FWE) are shown by the heat map, and in the difference map Δβ is further threshold at 0.4. VC activation generally increased the IC noise response. (**b**) Analysis ROIs defined in the external cortex of the IC (ECIC), dorsal cortex of the IC (DCIC) and the central nucleus of the IC (CNIC) (right side) as demarcated in Paxinos & Watson rat brain atlas (left side). The ECIC and CNIC ROIs contain voxels from both Bregma -9.1 mm and -8.1 mm, while the DCIC ROI from only Bregma -9.1 mm. (**c**) BOLD signal profiles extracted from the defined ROIs in the inferior colliculus (IC) and its subnuclei during the auditory stimulation (AUD; area in shade) without and with the 1 Hz VC blue light stimulation (Light). (**d**) Comparison between the BOLD responses (β values) to the broadband noise stimulation without and with VC blue light stimulation in each ROI across individual animals. Note that the solid colored line (blue or red) in each plot represents the averaged β value. No significant differences in IC noise response were observed. The results are presented as means ± standard error of the mean. Statistical comparisons were performed using paired two-sample t-test followed by Holm-Bonferroni correction with n.s. for not significant.

**
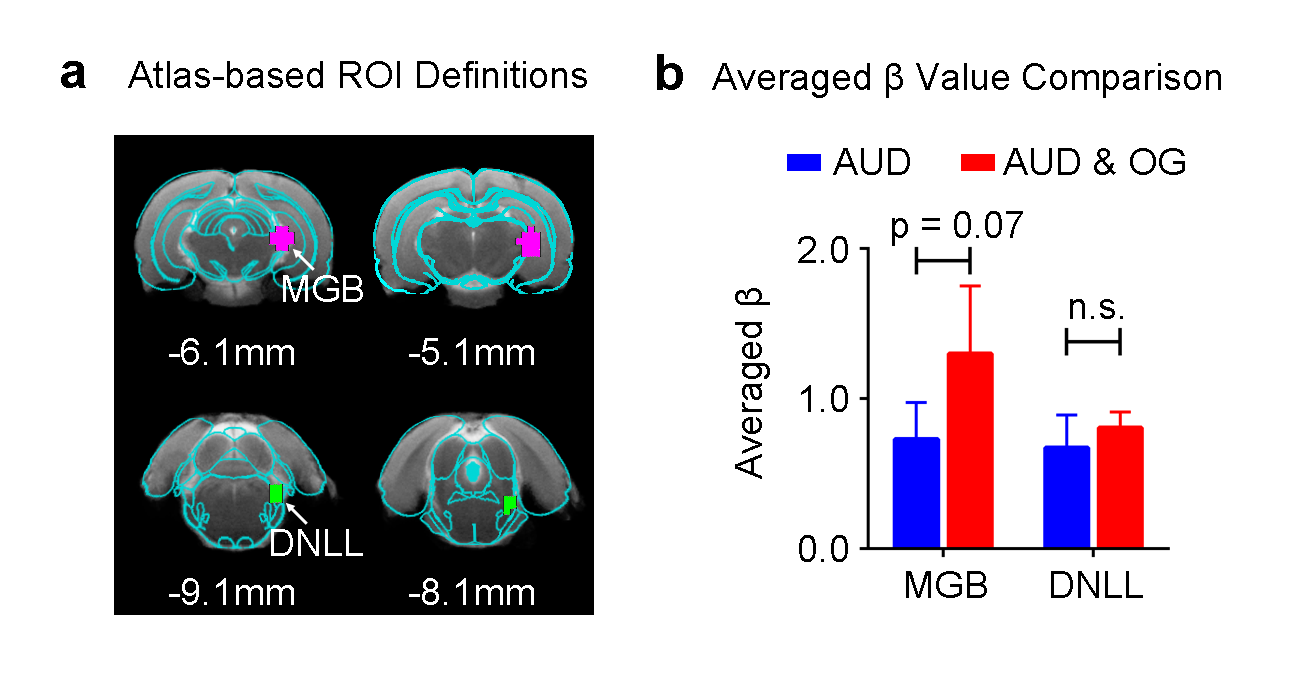
**

**Supplementary Figure S7 Influences of 10 Hz optogenetic stimulation in the VC on auditory fMRI responses in the medial geniculate body (MGB) and lateral lemniscus (LL).** (**a**) Analysis ROIs defined in the MGB and dorsal nucleus of the LL (DNLL) based on the Paxinos & Watson rat brain atlas. MGB response is not shown, as it is not significant after correction for FWE, and LL response to broadband noise stimulation is shown in Figure 4a. (**b**) Comparison between the BOLD responses (mean β values) to the auditory stimulation (AUD) without and with 10 Hz VC optogenetic stimulation (OG) in MGB and DNLL. The MGB response exhibited an increasing trend (p<0.05 for paired Student’s t test but does not pass Holm-Bonferroni correction). The DNLL response was not significantly influenced by the optogenetic stimulation. The results are presented as means ± standard error of the mean. Statistical comparisons were performed using paired two-sample t-test followed by Holm-Bonferroni correction. n.s. indicates not significant.
